# Supplementary material for: The Yeast Gsk-3 Kinase Mck1 Is Necessary for Cell Wall Remodeling in Glucose-Starved and Cell Wall-Stressed Cells
Source: Int J Mol Sci. 2025 Apr 9;26(8):3534. doi: 10.3390/ijms26083534 (PMC12027387; doi:10.3390/ijms26083534)
Supplement: Supplementary file 1 [file ijms-26-03534-s001.zip › Supplemental File S1.pptx]

## Slide 1
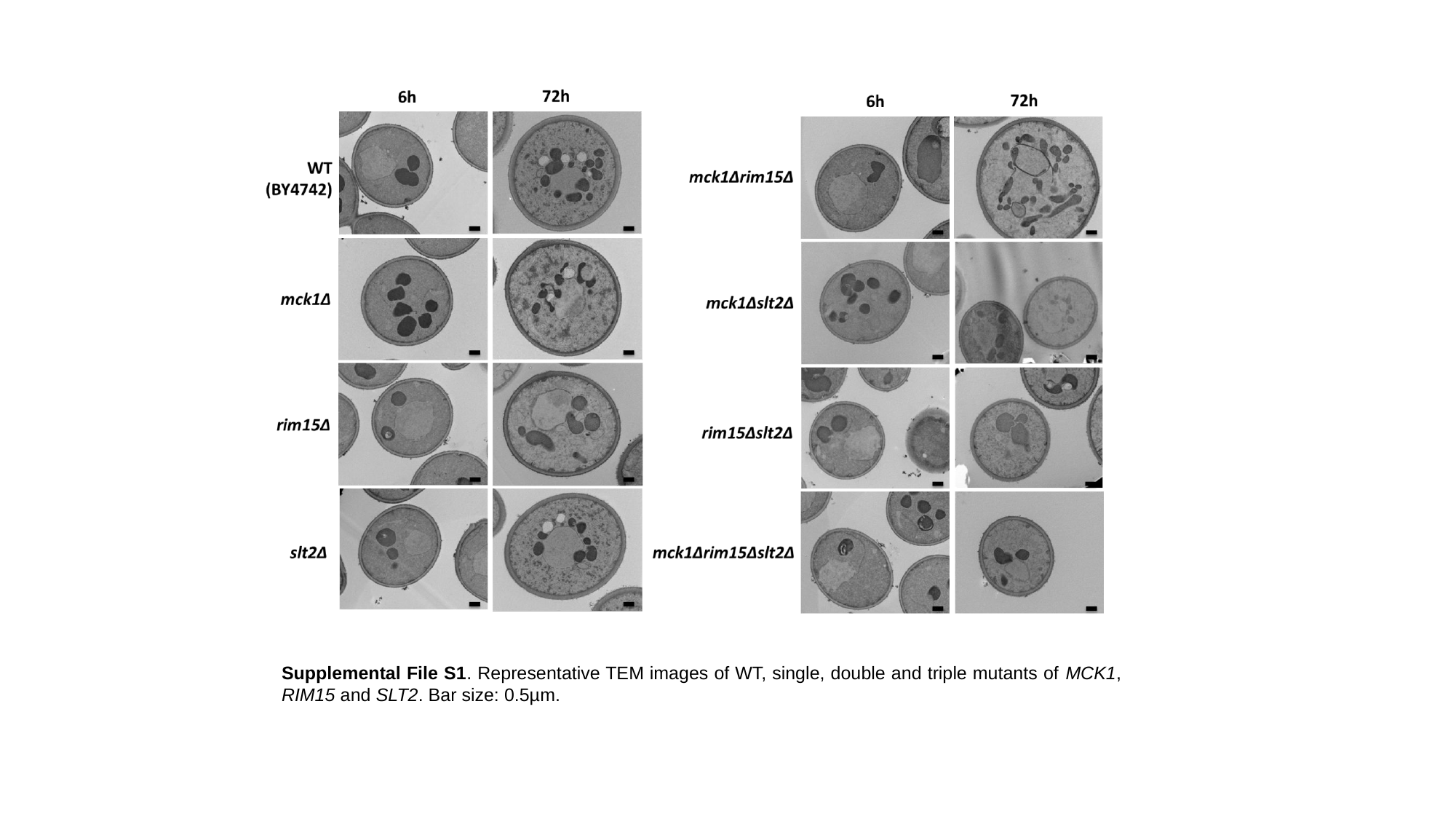

Supplemental File S1. Representative TEM images of WT, single, double and triple mutants of MCK1, RIM15 and SLT2. Bar size: 0.5µm.
